# Electrochemical characterization of TiO_2_/WO_x_ nanotubes for photocatalytic application

**DOI:** 10.1186/1556-276X-9-573

**Published:** 2014-10-14

**Authors:** Raman Vedarajan, Shoto Ikeda, Noriyoshi Matsumi

**Affiliations:** 1 Department of Materials Science, Japan Advanced Institute of Science and Technology, 1-1Asahidai, Nomi, Ishikawa 923-1292, Japan

**Keywords:** TiO_2_/WO_x_ nanotubes, Photoelectrochemical water splitting, Photocatalytic application

## Abstract

TiO_2_/WO_x_ nanotubes have unique photo-energy retention properties that have gathered scientific interest. Herein, we report the synthesis, morphological characterization, and the electrochemical characterization of TiO_2_/WO_x_ nanotubes compared with pure TiO_2_ nanotubes, prepared by anodization technique. Significant structural differences were not observed in TiO_2_/WO_x_ nanotubes as observed by using scanning electron microscopy and transmission electron microscopy. The charge transfer resistance of TiO_2_/WO_x_ before and after photo irradiation determined by using electrochemical impedance spectroscopy proves the inherent energy retention property which was not observed in pure TiO_2_ nanotubes.

## Background

Solar energy is clean, safe, and limitless; hence, tapping solar energy would be beneficial at global scale. In order to realize a solar-driven energy conversion device, semiconducting materials are required that absorb sunlight and accomplish an unhindered electron injection from valence band to conduction band, enabling performance of electric work in the circuit. Photoelectrochemical (PEC) water splitting to generate hydrogen for its use as fuel is considered to be a feasible alternative and sustainable energy system. Ideal materials for photoelectrochemical water splitting are semiconductors, which Fujishima and Honda first demonstrated using TiO_2_. As a result, this field of research has gathered significant attention by the research fraternity to achieve a highly efficient system producing hydrogen and oxygen by splitting up of water using most of the solar spectrum supplemented by a little or no electrical energy. Metal oxides, in particular, TiO_2_ and WO_3_, possess congenial electronic structure leading to good photoactivity and chemical stability. Further, their low cost and availability in abundance make them the chosen material for photoanodic reactions in aqueous electrolytes [1]. TiO_2_ has been a material extensively studied for water photooxidation [2]. More recently, TiO_2_ nanotubes (TNT) have gained much attention due to its simple synthesis [3] and enhanced photoelectrochemical performance over its nanoparticle counterpart. Despite the improved performance of TiO_2_ nanotubes, enhancing the visible light activity as well as reducing the charge recombination losses is required to increase water-splitting efficiency if practical applications are to be realized. On the other hand, tungsten trioxide (WO_3_) is a visible light photoactive material [4] with a bandgap of approximately 2.7 eV. Although it is a promising material by itself, coupling it with TiO_2_ has shown to be beneficial in many applications [5]. The formation of heterojunctions of two semiconductors is an appealing method to increase visible light activity while maintaining the properties of each component. Moreover, the incorporation of WO_3_ is, in particular, ambient due to the nearly similar ionic radius of W^+6^ to that of Ti^4+^. As a result of which WO_3_ can be easily coupled into the TiO_2_ lattice during anodization process. Titania nanotubes incorporated with tungsten oxide have been reported to possess enhanced optical and electronic properties compared to its pure form [6-14]. Further, this mixed oxide composite nanotube shows a unique photon energy retention feature, i.e., the photon to electrical energy conversion process does not cease immediately after curbing the photon influx but stops gradually. This feature in TiO_2_ + WO_x_ nanotubes has not been studied in depth. Investigations angled from the energy retention viewpoint of this metal oxide composite and its effect over dye-sensitized solar cell and photoelectrochemical water splitting was conceptualized in this endeavor.

Hence, in this present work, we have attempted to employ electrochemical impedance spectroscopy in evaluating TiO_2_/WO_x_ nanotubes as a candidate material for water splitting. The TiO_2_/WO_3_ nanotubular composite was prepared through a single-step anodization of titanium in an aqueous bath of NH_4_F and H_3_PW_12_O_40_ (phosphotungstic acid (PTA)). The morphology of the nanotubes was characterized using scanning electron microscopy (SEM), transmission electron microscopy (TEM), and energy dispersive X-ray (EDX) analysis. The nanotubes were tested for its charge transfer resistance in an electrolyte at pH approximately 6 under air mass (AM) 1.5 simulated solar irradiation. A time-oriented electrochemical impedance spectroscopy was carried out to assess the charge retention property of the mixed oxide nanotube and was compared with its pure counterpart.

## Methods

### Synthesis of TiO_2_/WO_x_ nanotubes

Synthesis of TiO_2_ nanotubes and TiO_2_/WO_x_ was carried out similar to previously reported procedure. In short, cleaned, polished titanium metal strips were anodized at DC voltage of 50 V for two and a half hour in an aqueous solution containing 0.5 wt.% NH_4_F for TNT synthesis and 0.5 wt.% NH_4_F with 2.4 wt.% PTA (Kanto Chemicals, Tokyo, Japan) for TNT-WO_x_ synthesis. Preliminary studies indicated an amount of 2.4 wt.% of PTA to be the optimal loading [6].

### Characterization of TNT and TNT/WO_x_

Firstly, the synthesized TiO_2_ and TiO_2_/WO_x_ nanotubes were characterized by using scanning electron microscope (SEM; Hitachi Model H-4600, Tokyo, Japan) and transmission electron microscope (TEM; Hitachi Model H-7100, Tokyo, Japan) for its morphology. Secondly, the elemental analysis was carried out using energy dispersive X-ray (EDX; Horiba, Kyoto, Japan) analysis. Finally, the electrochemical experiments were carried out in a conventional three-electrode setup in which the TiO_2_ or TiO_2_/WO_x_ served as the photoanode, Pt as the cathode, and Ag/AgCl as the reference electrode. The electrolyte used was 0.1 M NaOH (Kanto chemicals, Tokyo, Japan). A computer-controlled potentiostat coupled with a frequency response analyzer was used to control the potential and record the electrochemical impedance spectra (VersaStat-3, Princeton Applied Research, Oak Ridge, TN, USA). The photoanodes were illuminated by a 300 W solar simulator with an AM 1.5 at one sun intensity (Peccell Technologies, Inc., Yokohama, Japan) (approximately 87 mW/cm^2^).

## Results and discussion

### Morphological and elemental analysis

**Figure 1:**
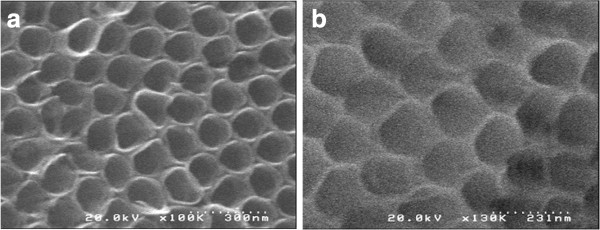
**SEM image of TiO**_**2 **_**and TiO**_**2**_**/WO**_**x **_**nanotubes. (a)** SEM image of TiO_2_ nanotubes **(b)** SEM image of TiO_2_/WO_x_ nanotubes.

**Figure 2:**
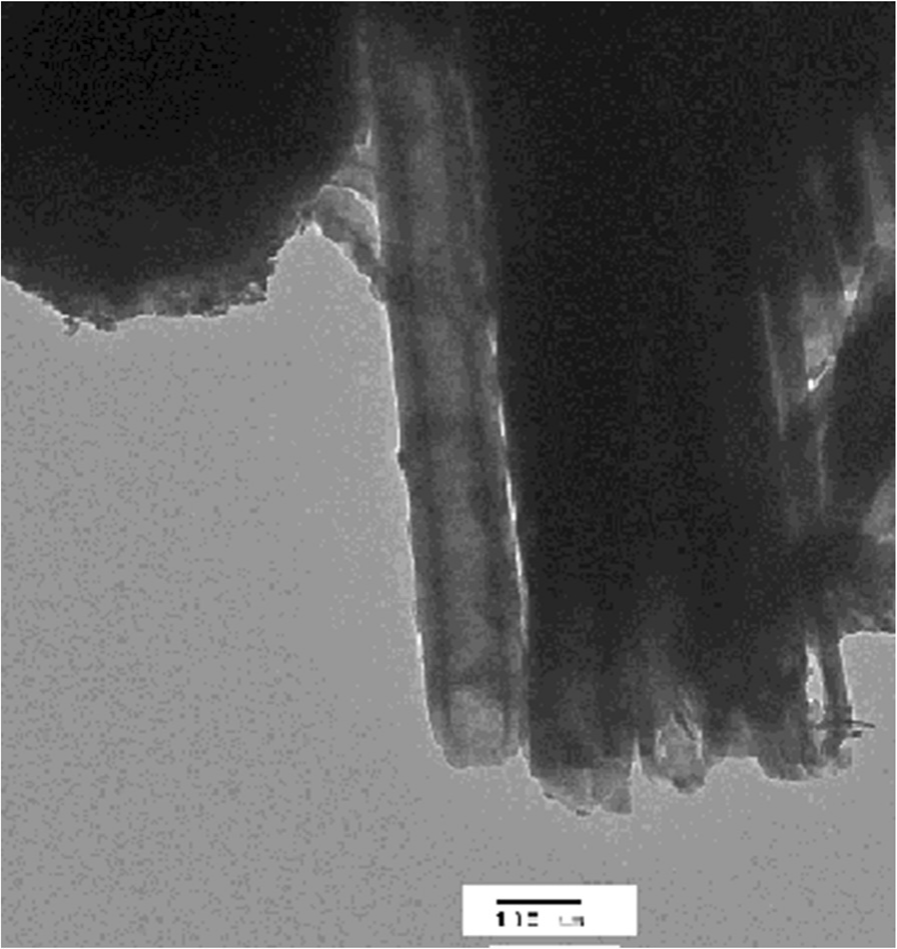
**TEM image of TiO**_**2**_**/WO**_**x **_**nanotubes.**

Scanning electron micrographs of TiO_2_ and TiO_2_/WO_x_ nanotubes showed that the morphology of both types were similar (Figure 1a,b). The honeycomb-like arrangement of the nanotubes remained unaffected by the presence of PTA during the anodization of Ti for the formation of TiO_2_/WO_x_. The pores were well formed and appeared uniformly circular. In both, the pure TiO_2_ nanotubes and one with WO_x_, the pore size was found to be in a range of 90 to 120 nm. Further, the same was confirmed from transmission electron micrographs (Figure 2). Also, in the case of the TEM micrographs, the samples anodized in the presence of PTA showed intermittent dark spots which can be attributed to the presence of a heavier element compared to Ti indicating the incorporation of WO_x_. Energy dispersive X-ray analysis was carried out to determine the elemental composition of the nanotubes and to confirm the incorporation of the W in the nanotubes. Peaks at around 2.5 eV and at 9 eV confirmed the presence of W. WO_x_ being the most stable form of W in an oxidizing medium, such as the one used in this study, and the presence of W can be ascribed to WO_x_.

### Electrochemical characterization

**Figure 3:**
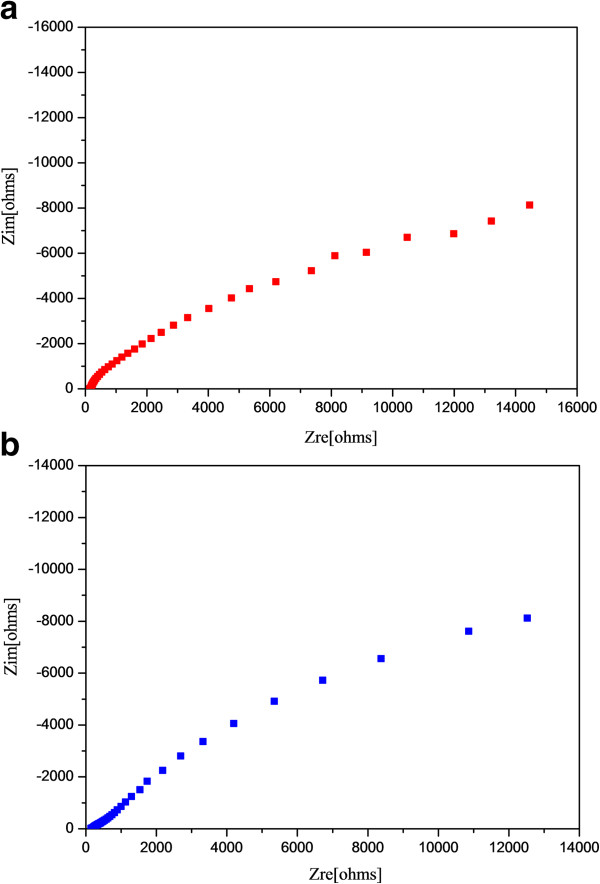
**Electrochemical impedance spectra of (a) TiO**_**2 **_**nanotubes (b) TiO**_**2**_**/WO**_**x **_**nanotubes in 0.1 M NaOH solution.**

A Nyquist plot at open-circuit potential condition of TiO_2_/WO_x_ and TiO_2_ in 0.1 M NaOH under AM 1.5 solar-simulated irradiation is given in Figure 3a,b. The nonlinear regression fitting using a conventional Randle's circuit (R(QR)); routine gave *R*_ct_ values of 1.48 × 10^4^ and 6.22 × 10^4^ Ω for TiO_2_/WO_x_ nanotubes and TiO_2_ nanotubes, respectively. A lower charge transfer resistance is indicative of recombination suppression by improved charge transport to the electrolyte. The improvement of the TiO_2_/WO_x_ system over TiO_2_ could be due to the formation of localized TiO_2_/WO_x_ heterojunctions or through the formation of W^6+^ surface states which act as mediators for charge transfer to the electrolyte.

**Figure 4:**
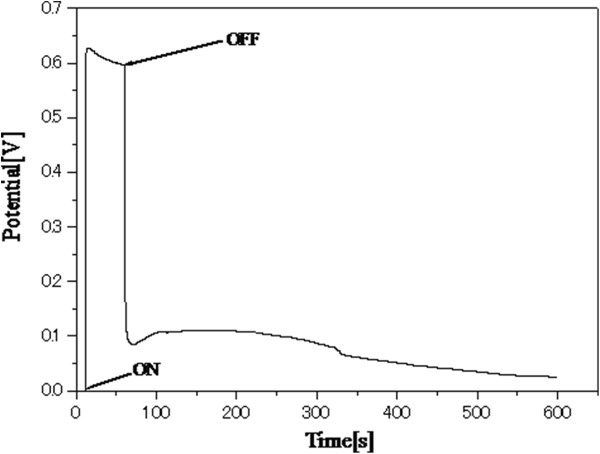
***V***_**oc **_**of TNT/WO**_**x **_**in a dye-sensitized solar cell (N719 dye) under 1.5 AM simulated solar irradiation.**

**Figure 5:**
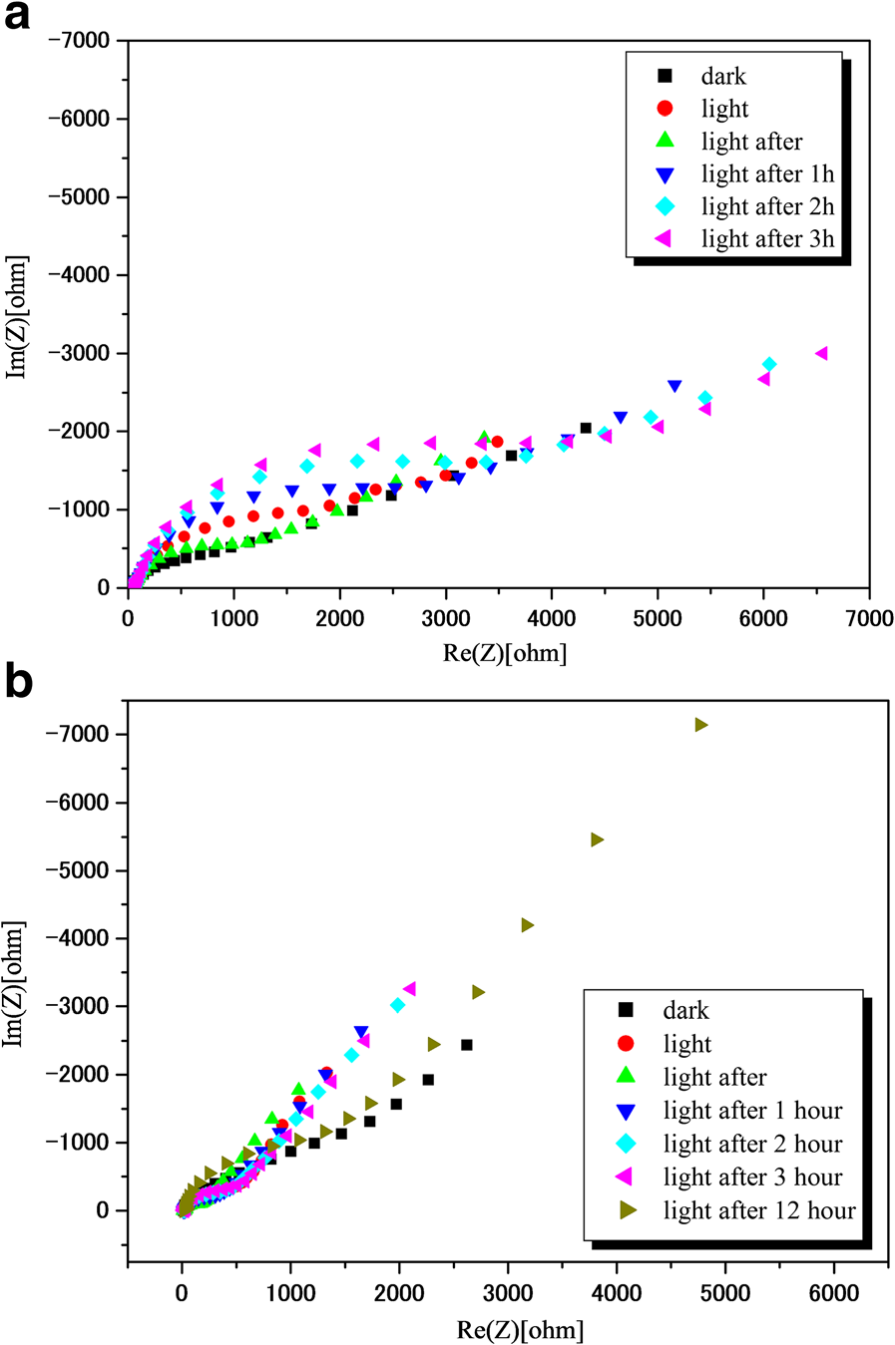
**Nyquist plots of (a) TiO**_**2 **_**nanotubes and (b) TiO**_**2**_**/WO**_**x **_**nanotube electrodes.** In 0.1 M NaOH under 1.5 AM simulated solar irradiation.

**Figure 6:**
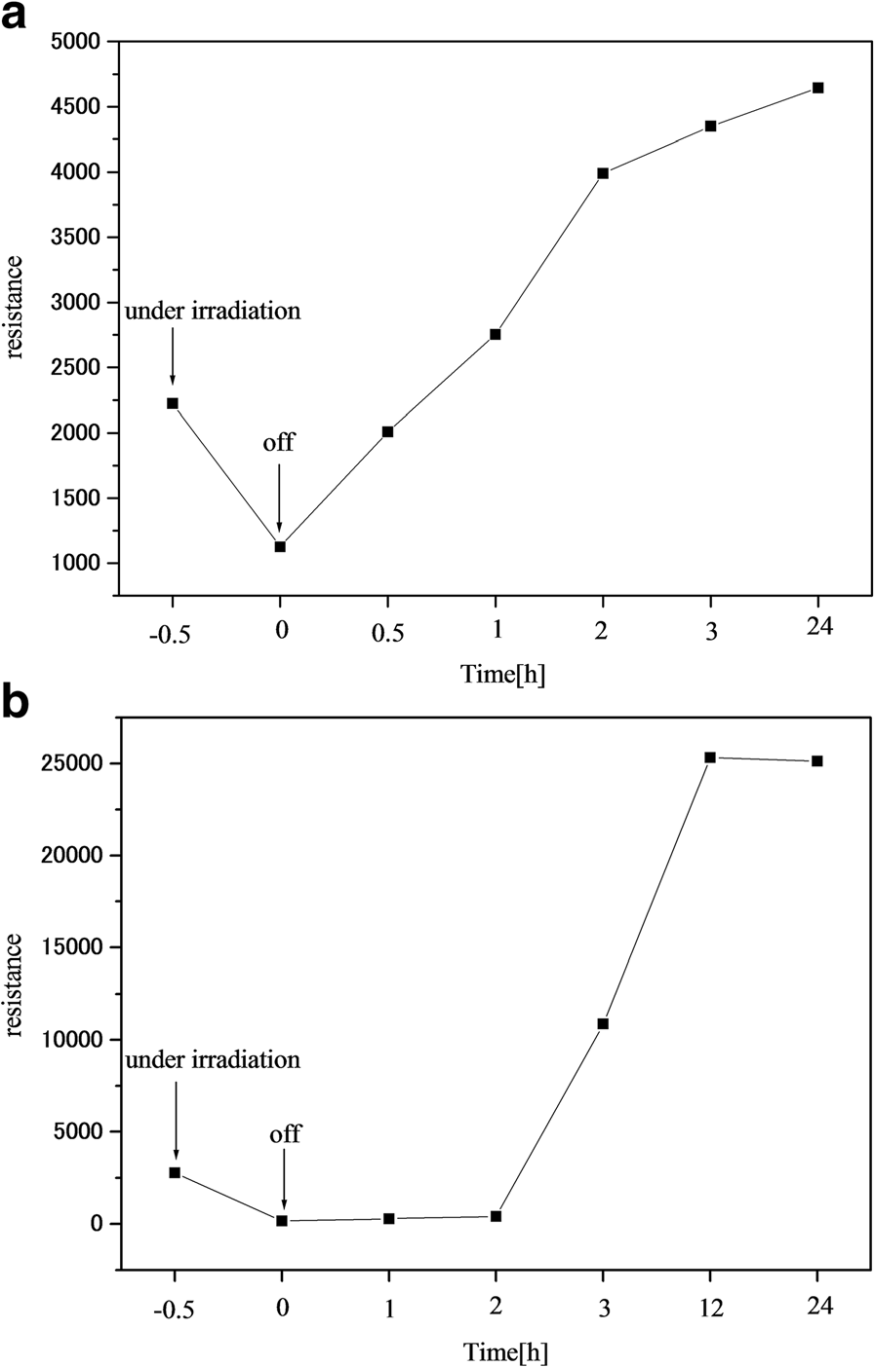
**Effect of time on impedance after photoirradiation. (a)** TiO_2_ nanotubes **(b)** TiO_2_/WO_x_ nanotubes obtained from Nyquist plots (Figure 5).

In order to determine the photovoltage of the semiconductor material, the open-circuit potential (*V*_oc_) of TiO_2_/WO_x_ electrodes were determined under photoirradiated condition as well as dark by constructing a conventional dye-sensitized solar cell in a sandwich-type configuration. The dye employed for this study was a commercially available N719 dye. As depicted in Figure 4, the photovoltage increased steeply with photoirradiation to a value of 0.73 V and later steadied at 0.71 V. However, on cutting photon influx by switching off the light source, the photovoltage initially dropped drastically to a value of 0.093 V. This was followed by an anomalous trend in the photovoltage, wherein the voltage showed an increasing trend for a few seconds and reached a plateau at 0.12 V and stayed at this potential for approximately 100 s before starting to fall continuously to 0 V. This behavior in TiO_2_/WO_x_ dye-sensitized electrode provoked further analysis to validate this behavior for other photocatalytic activity. Hence, a time-oriented electrochemical impedance spectroscopy was carried for both, the pure TiO_2_ nanotube electrode as well as the TiO_2_/WO_x_ nanotube electrode. The impedance measurements were carried out before the photoirradiation and at regular intervals for 12 h after photoirradiating the electrodes for 15 min under one sun condition. The results of the impedance measurements are presented in Figure 5a,b. All spectra were fitted with appropriate equivalent circuits. Although complicated systems like the present one are fitted with transmission-type circuits, in order to understand the overall impedance behavior of the material, the simpler Randle's circuit (R(QR)) or the modified Randle's circuit (R(Q(RW))) with diffusion parameter was used. However, the charge transfer resistance of the material will remain unaffected. All the charge transfer resistances are plotted against time as shown in Figure 6. Both the TiO_2_ nanotubes as well as the TiO_2_/WO_x_ electrodes exhibited decreased charge transfer resistance immediately after irradiation compared to the charge transfer resistance during irradiation. This can be attributed to the time lag involved in the excitation of the electron in to the conduction band, subsequent charge transfer from the electrolyte and the impedance measurement equilibrating time period. However, considering the constant error involved for both the systems, more significant observations were that the charge transfer resistance increased sharply under dark condition and attained a maximum within 3 h of curbing the photon influx in the case of the TiO_2_ nanotubes. On the other hand, in the case of TiO_2_/WO_x_ nanotubes, the increase in charge transfer resistance after cutoff of photoirradiation was not evident and the resistance remained unchanged at a very low value for a period of 3 h before showing an increasing trend. Further, the maximum resistance value was not attained before 12 h after curbing the photoirradiation. This phenomenon in the mixed oxide nanotube electrode can be attributed to the retention of energy by WO_x_ during high photon influx and releasing it during photon starvation condition. This attribute of TiO_2_/WO_x_ nanotubes could find potential application in photoelectrochemical water splitting.

## Conclusions

The morphological and elemental analyses were carried out by scanning electron microscopy and transmission electron microscopy. No significant structural difference was observed in TiO_2_/WO_x_ nanotubes compared to TiO_2_ nanotubes revealing honeycomb structure. Electrochemical impedance spectroscopy exhibited a high photoactivity from TiO_2_/WO_x_ nanotubes compared to TiO_2_ nanotubes. A time-dependent impedance analysis evinced the charge retention property of the mixed oxide, indicating its possible use in many photocatalytic applications.

## Competing interests

The authors declare that they have no competing interests.

## Authors' contributions

RV carried out the morphological studies, helped MO in performing electrochemical studies, and drafted the manuscript. SI carried out the electrochemical studies. NM conceived the study and helped to draft the manuscript. All authors read and approved the final manuscript.
